# Spectrum of Outpatient Illness in a School-Based Cohort in Haiti, with a Focus on Diarrheal Pathogens

**DOI:** 10.4269/ajtmh.14-0059

**Published:** 2015-04-01

**Authors:** Valery E. M. Beau De Rochars, Meer T. Alam, Taina Telisma, Roseline Masse, Sonese Chavannes, Marie G. Anilis, Herold Jean Guillaume, Gedeon Gelin, Ericka L. Kirkpatrick, Bernard A. Okech, Thomas A. Weppelmann, Mohammed Rashid, Stephanie Karst, Judith A. Johnson, Afsar Ali, J. Glenn Morris

**Affiliations:** Emerging Pathogens Institute, University of Florida, Gainesville, Florida; Department of Health Services Research, Management and Policy, College of Public Health and Health Professions, University of Florida, Gainesville, Florida; Department of Environmental and Global Health, College of Public Health and Health Professions, University of Florida, Gainesville, Florida; Christianville Foundation Boulevard Marechal/Ruelle Christianville, Gressier, Haïti; Centre Haïtien de Recherche en Sciences de la Santé, Saint Louis du Nord, Haiti; Department of Molecular Genetics and Microbiology, College of Medicine, University of Florida, Gainesville, Florida; Department of Pathology, Immunology, and Laboratory Medicine, College of Medicine, University of Florida, Gainesville, Florida; Department of Medicine, College of Medicine, University of Florida, Gainesville, Florida

## Abstract

Currently, there are only limited data available on rates of major diagnostic categories of illnesses among Haitian children. We have established a cohort of 1,245 students attending schools run by the Christianville Foundation in the Gressier/Leogane region of Haiti, for whom our group provides primary medical care. Among 1,357 clinic visits during the 2012–2013 academic year, the main disease categories (with rates per 1,000 child years of observation) included acute respiratory infection (ARI) (385.6 cases/1,000 child years of observation), gastrointestinal complaints (277.8 cases/1,000 child years), febrile illness (235.0 cases/1,000 child years), and skin infections (151.7 cases/1,000 child years). The most common diarrheal pathogen was enteroaggregative *Escherichia coli* (present in 17% of children with diarrhea); *Vibrio cholerae* O1 and norovirus were the next most common. Our data highlight the importance of better defining etiologies for ARI and febrile illnesses and continuing problems of diarrheal illness in this region, including mild cases of cholera, which would not have been diagnosed without laboratory screening.

## Introduction

In 2010, Haiti faced two major events that had a serious impact on the health of the population. This included the devastating earthquake that struck Haiti in January 12, which resulted in the death of some 220,000 people and led to the internal displacement of > 1.5 million persons, followed in October 2010 by a large cholera outbreak that started in the Artibonite Department and spread rapidly throughout the country.^1^–^3^ Both events resulted in major mobilization by relief agencies, non-governmental organizations (NGOs), and the international community. However, now, 4 years after the emergencies, most of these organizations have withdrawn, leaving behind a number of questions about the relative incidence of various disease entities and appropriate prioritization of health-care resources for the Haitian population.

**Figure 1.**
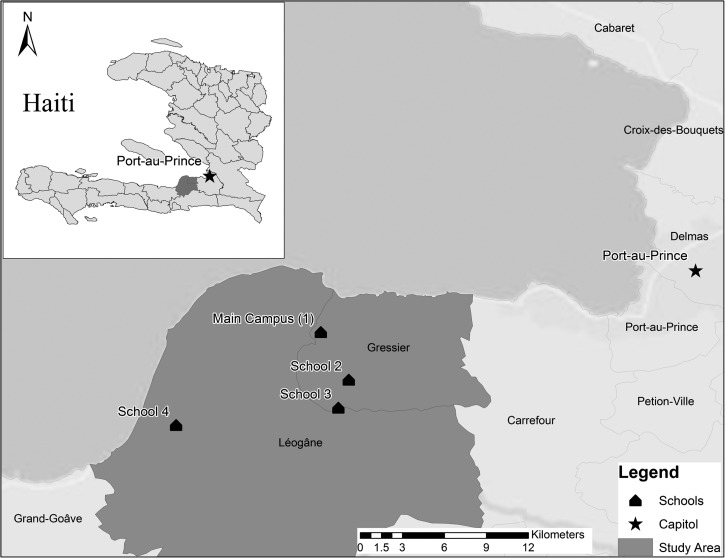
Christianville school network in Gressier and Leogane, Haiti, 2013.

Our group at University of Florida (UF) has established a system of clinical surveillance in a semi-urban/rural area near Gressier (located 12 miles southwest of the capital Port-au-Prince), which makes use of the Christianville Foundation school network (Figure 1; Table 1). To develop preliminary incidence data for major disease categories (accompanied by a more detailed analysis of diarrheal pathogens), information was collected for all school clinic consultations during the academic year from October 2012 to June 2013, from a cohort of 1,245 students aged 3–27 years.

As in many parts of Haiti, free public education opportunities are limited in this region, with most children attending private school; in keeping with this pattern, students at the Christianville schools are charged tuition, but on a sliding scale, with a third or more paying only minimal tuition or on full scholarships. In general, the smaller schools in the Christianville system (schools 2–4, Figure 1), which only go through grade 6, are located in resource-poor areas, with limitations in safe water and sanitation infrastructure and availability of health centers. In an initial needs assessment, which included a 10% random sample of all student households, average household size was 7 people, 74% of households had a latrine, and wells were the main water source used by 58% of households; 97% of households had access to a cellular phone.

**Figure 2.**
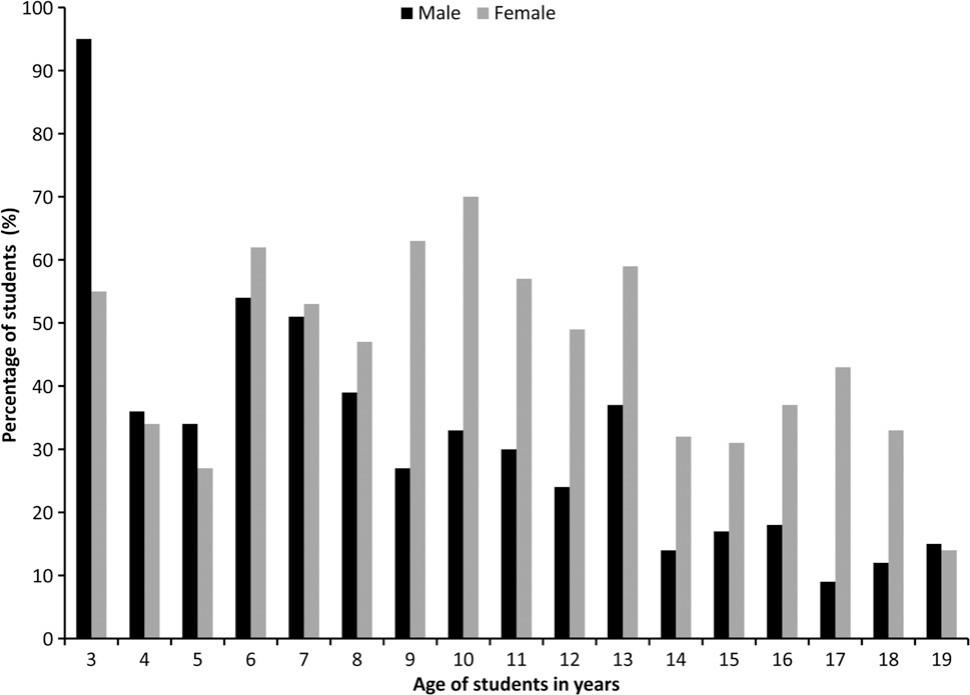
Breakdown of age of students by gender visited the clinic during the 2012–2013 academic year, Gressier, Haiti.

The school clinic is staffed by a full-time physician and two full-time nurses. Every presenting child at the clinic was counted as a new case. Additional visits with the same complaint within 2 weeks of an initial visit were not counted as new cases; however, students with the same complaint outside of this therapeutic window were counted as new cases. A total of 1,357 new cases were recorded for the 2012–2013 academic year (Figure 2). November had the most new cases; March and December had vacation periods, and consequently the number of cases in these months was lower. Almost 20 disease affections or types were recorded during clinical visits. De-identified case data were provided by the school clinic staff for retrospective analysis after signing of requested confidential agreement documents, as approved by the UF Institutional Review Board (IRB) and the Haitian National IRB (National Bioethics Committee). The most common complaints are shown in Table 2, together with data on case numbers and rates per 1,000 child years of observation.

Respiratory complaints were most common, with a rate of 385.6 cases per 1,000 child years of observation (Table 2). Acute respiratory infections (ARIs) were defined as any child presenting with cough, respiratory symptoms, and fever (temperature > 37.9°C). Within ARI, upper respiratory infections were defined by rhinitis and/or pharyngitis, with no evidence of lung involvement; lower respiratory infections were defined based on the presence of lung findings, including labored breathing, rales and/or stridor, and were generally treated with antibiotics. The predominance of lower respiratory infections in our clinic population may reflect, to a degree, a self-selection process, with children with milder upper respiratory infections not seeking medical care. We did not have access to culture or polymerase chain reaction (PCR)–based diagnostic modalities, so cannot comment on possible etiologic agents in these cases. Asthma as a long-term respiratory affliction was defined separately as wheezing, breathlessness, and coughing with no fever. We found asthma to be relatively uncommon in our student population (7.5 cases/1,000 child years), in keeping with prior observations that asthma rates are low in developing countries.^4^

Gastrointestinal complaints were seen with a rate of 277.8 cases per 1,000 child years of observation. Gastrointestinal complaints were divided into three general syndromes, including 1) abdominal/periumbilical discomfort and/or anal pruritus, without diarrhea; 2) nausea/gastritis, some vomiting but without diarrhea; or 3) gastroenteritis, defined as diarrhea with or without vomiting. Efforts were made to collect stool samples from all students with diarrhea or possible intestinal infections, with samples sent within 2 hours for analysis to the UF field microbiology laboratory adjacent to the main school campus in Gressier. On receipt in the laboratory, samples were immediately processed for pathogens, including *Shigella*, *Salmonella*, *Vibrio*, *Aeromonas*, *Campylobacter*, and diarrheagenic *E. coli* following standard microbiological procedures.^5^–^7^ Laboratory methods are described in greater detail in the supplement accompanying this article.

Forty-one (93%) of 44 students with diarrhea had stool samples submitted. Among students without diarrhea, 124 had stool samples submitted. Among those with diarrhea, 54% had a potential pathogen identified (Table 3) versus 13% of children without diarrhea. Enteroaggregative *E. coli* (EAgEC) was the most common pathogen identified; EAgEC was also a common isolate from normal stool samples, although its isolation was significantly associated with diarrhea. Toxigenic *V. cholerae* O1 was isolated in three cases: two were in students who presented with mild diarrhea with no history of vomiting, fever, and no hospitalization, and the third case patient was hospitalized for severe diarrhea. These observations reinforce the concept that the majority of cases of cholera involve mild to moderate diarrhea and may not be recognized without further diagnostic evaluation. Results are also in keeping with the observation that 75% of persons seropositive for cholera in a study conducted in Grand Saline (a community in the Artibonite Department, the epicenter for the epidemic) some 6 months after the onset of the epidemic gave no history of attendance at a cholera clinic or occurrence of diarrheal disease.^8^

We did not screen for rotavirus as we were evaluating older children; based on results from a smaller recent study from Haiti, it is possible that rotavirus accounted for some of the diarrheal cases for which we did not identify an etiology. We did screen for cryptosporidium, but did not identify any cases; however, we screened visually, rather than by enzyme-linked immunosorbent assay (ELISA) (as was done in the GEMS study), and consequently may have missed some cases. Norovirus and *Giardia* were identified in a small percentage of patients with diarrhea, but were also common in samples from patients without diarrhea, suggesting that their identification in a stool sample should not be assumed to indicate an etiologic role in disease.

Febrile illnesses without an obvious source of infection constituted the third most common presenting complaint, accounting for 235 cases/1,000 child years or 16.2% of all cases seen at the clinic. All case patients presented at the clinic with a temperature of at least 38°C in the absence of ARI symptoms or other obvious sources of fever. In some cases, clinic staff, based on clinical suspicion, submitted blood samples to an outside commercial laboratory for screening for malaria and typhoid. Eight samples came back positive for malaria and 10 with serologic results (Widal) suggestive of typhoid fever. Facilities were not available for collection and analysis of blood cultures.

**Figure 3.**
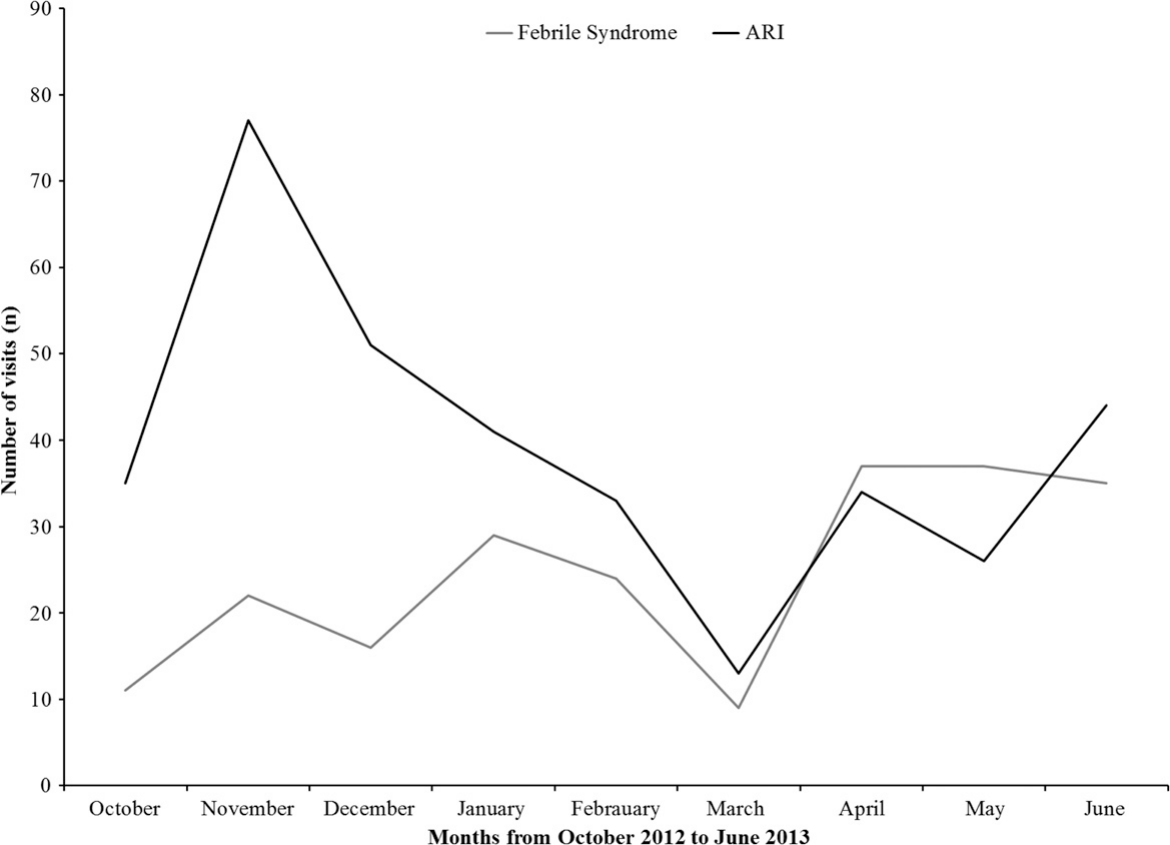
Monthly comparison between febrile syndrome and acute respiratory illness (ARI) at the Christianville school clinic for the 2012–2013 academic year.

In contrast to the ARI cases, which peaked in the fall/winter season, these cases appeared to have more of a spring/summer peak (Figure 3). In developing countries, the differential for such cases usually includes both malaria and typhoid, with empiric therapy for one, or both, often prescribed. Because of technical difficulties in the commercial laboratory that we were using, results were not obtained for all suspect malaria cases for which samples were submitted; given this problem, combined with the fact that only a small subset of fever patients were screened for malaria, there are likely to have been unidentified malaria cases in our cohort. However, even if we assume that we identified only a portion of cases, it is unlikely that malaria was responsible for the bulk of the cases of unexplained fever seen in the clinic. Support for this observation comes from a serologic study conducted in the spring on a random sample of 510 asymptomatic children in the cohort, in which approximately 12.4% (95% confidence interval [CI]: 9.4–15.2) had evidence of previous exposure to *Plasmodium falciparum*.^9^ We did not screen for dengue in this cohort; however, a recent serosurvey conducted on a serum collected from a subset of children from this cohort (*N* = 582) indicated the presence of antibodies to dengue virus in 74% of children (95% CI: 70.5–77.6) and with little evidence of West Nile virus antibodies present in children from this cohort. This is in agreement with a recent Centers for Disease Control and Prevention (CDC) study in this region, which sampled expatriate and Haitian NGO workers, which suggests that there are high rates of dengue transmission.^10^ Recent studies from the United States also suggest that viral etiologies for fever cases are common, with the same viral groups noted for ARIs, as well as pathogens such as human herpes virus 6.^11^

Incidence of skin infections was 151.7 cases per 1,000 child years (Table 2). Primary clinical diagnoses included impetigo 30%, tinea capitis and corporis that accounted for 22% and pityriasis versicolor for 6%. In developing countries, those skin infections are highly prevalent among children, with impetigo the most common diagnosis.^12^,^13^ These lesions are “neglected” among tropical diseases in Haiti because they are not part of the reportable disease list and are not in the current strategic planning. There is a similar lack of data from the entire Caribbean, with no reports on the magnitude of these infections in this region.

## Comment

Our studies in the Christianville schools provided an opportunity to assess rates of major disease categories in a single semi-urban region in Haiti. As the school clinic only saw children enrolled in the school system, we have a denominator. As service was provided free of charge, and many, if not most, of the children did not have an alternative source of medical care, it is likely that we captured a majority of cases; however, there is the possibility of “leakage” from the system, particularly during holiday periods when clinic care was available, but may have been inconvenient as children were not physically present in school. Because of this, rates should be regarded as the lower end of a range, rather than as an absolute value. Rates are reported as cases per 1,000 child years of observation; although these rates approximate annual incidence rates per 1,000 children, they may differ slightly as our data were collected only for the 9-month academic year, resulting in possible overestimates of annual rates of ARI and other illnesses that have a winter peak, and undercounting of illnesses with summer peaks.

National data on various disease entities are reported by the Haitian Ministry of Public Health and Population (MSPP), but only as percent of total cases seen in clinic; the last census done in Haiti was in 2003 and since then, all public health intervention and implementation are based on projection from that census data, making it difficult to come up with accurate denominators. Subsequent natural disasters, including the 2010 earthquake, resulted in substantial population shifts, making data accuracy even more problematic. National data that are available have been reported through the Morbidity, Mortality, and Usage of Service Survey (EMMUS-V 2012), as well as the strategic planning process of MSPP.^14^,^15^ Numbers from these surveys reported only for children less than 5 years are included in Table 2. Our surveillance data (more recent, but from a single geographic region) present a somewhat different picture than that seen in the national data, with acute respiratory illness (ARI) more common and diarrheal illness playing a less important role.

Our goal in this report was to identify major disease categories in our student population to prioritize areas for further study and allocation of diagnostic and therapeutic resources. Where we found the greatest need was for diagnostic data for the major disease categories of ARI (for both upper and lower tract disease) and febrile illness with no obvious source. In the absence of such diagnostic data, particularly for febrile illness, there remains a strong tendency among clinicians to empirically treat for conditions such as malaria, which is likely to result in inappropriate therapy in a high percentage of children. Further studies are underway at our school clinics to improve diagnostic capacity, particularly for patients with these clinical entities. Such diagnostic testing is possible because of the associated UF field microbiology laboratory at the site. However, outside of a very small number of clinical laboratories, such testing is generally not available in Haiti. As efforts are made to decrease the country's overall disease burden, it is essential that diagnostic capabilities for these clinical categories be expanded to facilitate identification of key diseases, appropriate targeting of resources, and development of interventions.

## Figures and Tables

**Table 1:** Number of students enrolled by age group and by grade at the Christianville school in Gressier, Haiti, from October 2012 to July 2013, and number of visits seen by gender and by grade at the Christianville school clinic in Gressier, Haiti, from October 2012 to July 2013

Grade	Age group	Schools						Number of visits by gender			
		1	2	3	4	Total	%	Female	Male	Total	%
Kindergarten	3–5	133	66	47	40	286	23	118	167	285	21
Primary	6–11	330	94	90	77	591	47	337	224	561	41
Secondary	12–27	368	—	—	—	368	30	335	176	511	38
Total		831	160	137	117	**1,245**		**790**	**567**	**1,357**	

The bold numbers indicate the totals.

**Table 2:** Case number and rate per 1,000 child years of observation for disease syndromes seen at Christianville school clinic during the academic year 2012–2013, together with case percentage for major clinical syndromes and the corresponding national case percentages

Clinical diagnoses		Student age group (number of students)								Percentage of total clinic visits	National % of total clinic visits
		Kindergarten (286)		Primary (591)		Secondary (368)		Total (1,245)			
		Cases	Rate*	Cases	Rate	Cases	Rate	Cases	Rate		
Respiratory	Upper ARI	27	124.6	47	105.8	50	180.7	124	132.5		
	Lower ARI	72	334.8	103	231.8	55	198.8	230	245.7		
	Asthma	2	9.3	4	9.0	1	3.6	7	7.5		
	Total respiratory	**101**	**469.7**	**154**	**346.6**	**106**	**383.1**	**361**	**385.6**	**26.6**	**14.4**§
GI	Diarrhea	8	37.2	19	42.8	17	61.4	44	47.0		
	Intestinal Infections†	30	139.5	93	209.3	42	151.8	165	176.3		
	Gastritis‡	1	4.7	14	31.5	36	130.1	51	54.5		
	Total GI	**39**	**181.4**	**126**	**283.6**	**95**	**343.3**	**260**	**277.8**	**19.2**	**21**§
Febrile illness		**60**	**279.0**	**103**	**231.8**	**57**	**206.0**	**220**	**235.0**	**16.2**	**27**§
Skin infections		**43**	**200.3**	**64**	**144.0**	**35**	**126.5**	**142**	**151.7**	**10.5**	**3.9**∥
Trauma		11	51.2	27	60.8	32	115.7	70	74.8		
Headache		5	23.3	22	49.5	32	115.7	59	63.0		
Dysmenorrhea		0	0.0	1	2.3	56	202.4	57	60.9		
GU		4	18.6	21	47.3	31	112.0	56	59.8		
Anemia		7	32.6	19	42.8	29	104.8	55	58.8	4.1	9∥
Other	Sinusitis	0	0.0	0	0.0	1	3.6	1	1.1		
	Measles	0	0.0	0	0.0	1	3.6	1	1.1		
	Hemorrhoids	0	0.0	0	0.0	2	7.2	2	2.1		
	Epilepsy	0	0.0	0	0.0	3	10.8	3	3.2		
	Osteochondritis	0	0.0	0	0.0	2	7.2	2	2.1		
	Allergies	0	0.0	4	9.0	3	10.8	7	7.5		
	Tonsillitis	5	23.3	14	31.5	19	68.7	38	40.6		
	Conjuctivitis	1	4.7	0	0.0	0	0.0	1	1.1		
	Otitis	4	18.6	9	20.3	8	28.9	21	22.4		
	Arthritis	0	0.0	0	0.0	1	3.6	1	1.1		
	Total other	10	46.5	27	60.8	40	144.6	77	82.3		
Total clinical consultations		**280**		**564**		**513**		**1,357**			

The bold numbers indicate the main diseases seen.

ARI = acute respiratory illness; GI = gastrointestinal; GU = genitourinary.

* Rate calculated as number of cases per 1,000 child years of observation.

† Intestinal infections including abdominal pain without diarrhea.

‡ Gastritis includes nausea and gastritis without diarrhea.

§ EMMUS-V reported data.

∥ MSPP 2004 reported data.

**Table 3:** Distribution of enteric pathogens in diarrheal and nondiarrheal samples collected from children at Christianville school clinics in Gressier, Haiti, from October 2012 to July 2013

Name of identified pathogens	Total no. of samples (%) positive from children		*P* value*
	With diarrhea (*N* = 41)	Without diarrhea (*N* = 124)	
*Vibrio cholerae* O1 Ogawa	3 (7.3)	0 (0)	0.01
Nontyphoidal *Salmonella* spp.	2 (4.9)	0 (0)	NS (0.06)
*Campylobacter* spp.	1 (2.4)	0 (0)	NS
Enterotoxigenic *E. coli*	1 (2.4)	0 (0)	NS
Enteropathogenic *E. coli*	2 (4.9)	2 (1.6)	NS
Enteroaggregative *E. coli*	7 (17.1)	5 (4)	0.01
*Giardia* spp.	2 (4.9)	5 (4)	NS
*Entamoeba histolytica*	1 (2.4)	0 (0)	NS
Noro virus	3 (7.3)	4 (3.2)	NS
No pathogen identified	19 (46.3)	108 (87.1)	

NS = not significant at 0.05 level.

* Two-tailed Fisher's exact test.
